# Acknowledgement of manuscript reviewers 2015

**DOI:** 10.1186/s40201-016-0247-x

**Published:** 2016-02-22

**Authors:** Simin Nasseri, Amir Hossein Mahvi, Mitra Gholami, Nematallah Jaafarzadeh, Mohammad Reza Monazzam, Ramin Nabizadeh Nodehi, Kazem Naddafi, Noushin Rastkari, Roshanak Rezaei, Kamyar Yaghmaeian, Masud Yunesian

**Affiliations:** Tehran University of Medical Sciences, Tehran, Iran; Ahvaz Jondishapur Jondishapur University of Medical Sciences, Ahvaz, Iran

## Abstract

The editors of *Journal of Environmental Health Science & Engineering* would like to thank all our reviewers who have contributed to the journal in Volume 13 (2015).

**Amr Abdelkader**

Egypt

**Mehrnoosh Abtahi**

Iran

**Irshad Ahmad**

Saudi Arabia

**Ehsan Ahmadi**

Iran

**Farhang Akbar-Khanzadeh**

USA

**Mahmood Alimohammadi**

Iran

**Vali Alipour**

Iran

**Hoda Amiri**

Iran

**Syam Andra**

USA

**Mohsen Arbabi**

Iran

**Zahra Asadgol**

Iran

**Fatemeh Asgharzadeh**

Iran

**Vaishali Ashok**

India

**Seyed Davoud Ashrafi**

Iran

**Hasan Aslani**

Iran

**Ali Assadi**

Iran

**Zahra Atafar**

Iran

**Ali Azari**

Iran

**Gasem Azarian**

Iran

**Hulusi Barlas**

Turkey

**Edris Bazrafshan**

Iran

**Tim Cavileer**

USA

**Alireza Chackoshian Khorasani**

Iran

**Arash Dalvand**

Iran

**Nevzat Damla**

Turkey

**Mojtaba Davoodi**

Iran

**Mansooreh Dehghani**

Iran

**Emad Dehghanifard**

Iran

**Reza Dehghanzadeh**

Iran

**Ramazan Ali Dianati Tilaki**

Iran

**Sina Dobaradaran**

Iran

**Mohammad Hassan Ehrampoush**

Iran

**Mojtaba Ehsanifar**

Iran

**Mohammad Mahdi Emamjomeh**

Iran

**Akbar Eslami**

Iran

**Steve Evans**

UK

**Reza Faghihi**

Iran

**Mohammad Ali Faramarzi**

Iran

**MA Faramarzi**

Iran

**Samanesh MB Fard**

Australia

**Mahdi Farzadkia**

Iran

**Carlos Garcia**

Spain

**Mohammad Ghanbari**

Iran

**Farshid Ghanbari**

Iran

**Mohammad Taghi Ghaneian**

Iran

**Mitra Gholami**

Iran

**Fathhollah Gholami Borujeni**

Iran

**Akbar Gholampour**

Iran

**Rostam Golmohammadi**

Iran

**Abooali Golzary**

Iran

**Hassan Gomaa**

Canada

**Joe Griffitt**

USA

**Gilles Guibaud**

France

**Shi-Lun Guo**

China

**Vk Gupta**

India

**Anup Gurung**

Korea, South

**Sharifah BA Hamid**

Malaysia

**Darryl Hawker**

Australia

**Mohsen Heidari**

Iran

**Vahid Hosseini**

Iran

**Malcolm Hudson**

UK

**Jalil Jaafari**

Iran

**Ali Jafari**

Iran

**Akram Jamal**

Iran

**Mehran Javanbakht**

Iran

**Allahbakhsh Javid**

Iran

**Ahmad Jonidi**

Iran

**Sahand Jorfi**

Iran

**James Joseph**

India

**Babak Kakavandi**

Iran

**Kurunthachalam Kannan**

USA

**Ilgi Kapdan**

Turkey

**Anna Karakatsani**

Greece

**Behrooze Karimi**

Iran

**Hamid Karyab**

Iran

**Homa Kashani**

Iran

**Mohammad Khazaei**

Iran

**Hassan Khorsandi**

Iran

**Sonia Khoufi**

Tunisia

**Ali Koolivand**

Iran

**George Kyzas**

Greece

**Habib Langar**

Tunisia

**Luc Letenneur**

France

**Shuzhou Li**

Singapore

**Tayebe Bagheri Lotfabad**

Iran

**Hanyu Ma**

USA

**Nosrat Madadi Mahani**

Iran

**Amir Hossein Mahvi**

Iran

**Mohammad Malakootian**

Iran

**Afshin Maleki**

Iran

**M Maroosi**

Iran

**Mohammad Maroosi**

Iran

**Mohammad Reza Mehrasbi**

Iran

**Stephanie Melching-Kollmuss**

Germany

**Marino Menozzi**

Switzerland

**Izabela Michalak**

Poland

**Hamed Mohammadi**

Iran

**Ghasemali Mohebali**

Iran

**Fariba Mohsenzadeh**

Iran

**Mehdi Mokhtari**

Iran

**Ahmad Mokhtari**

Iran

**Mohammad Reza Monazzam**

Iran

**Deok Hyun Moon**

Korea, South

**Masoud Moradi**

Iran

**Masaaki Morikawa**

Japan

**Smj Mortazavi**

Iran

**Seyed Mohammad Mousavi**

Iran

**Gholamreza Moussavi**

Iran

**Ramin Nabizadeh Nodehi**

Iran

**Mahdiyeh Naderzadeh**

Iran

**Mahabir Nain**

India

**Jacek Namiesnik**

Poland

**Simin Nasseri**

Iran

**Parvin Nassiri**

Iran

**Balasubramanian Natesan**

Portugal

**Chelliah Navin**

USA

**Jose A Orosa**

Spain

**Murugesan Palanivel**

India

**A Panico**

Italy

**Yongzhen Peng**

China

**Mohammad Rafiee**

Iran

**Zahra Rahmani**

Iran

**Bahman Ramavandi**

Iran

**Noushin Rastkari**

Iran

**Madhumita Ray**

Canada

**Maryam Reza**

Canada

**Abbas Rezaee**

Iran

**Reza Rezaee**

Iran

**Aliakbar Rodbari**

Iran

**Dominik Roth**

Austria

**Aliakbar Roudbari**

Iran

**Imanol Ruiz-Zarzuela**

Spain

**Gasaymeh S**

Jordan

**Shahram Sadeghi**

Iran

**Reza Saeedi**

Iran

**Gholamhossein Safari**

Iran

**Maryam Sarkhosh**

Iran

**Devendra Saroj**

UK

**B Shahmoradi**

Iran

**Mansour Shamsipour**

Iran

**Asheesh Sharma**

India

**Guodong Sheng**

China

**Mehdi Shirzad-Siboni**

Iran

**Manzarbanoo Shojaeifard**

Iran

**Suntud Sirianuntapiboon**

Thailand

**Yufang Song**

China

**Mohammad Hossein Sowlat**

Iran

**Jörg Spitz**

Germany

**Delia Teresa Sponza**

Turkey

**Ghassan Mohammad Sulaiman**

Iraq

**Emanoil Surducan**

Romania

**Hassan Taghipour**

Iran

**George Tsiamis**

Greece

**Ioanna Vasiliadou**

Greece

**Josef Velisek**

Czech Republic

**Balaiya Velmurugan**

USA

**Manouchehr Vossoughi**

Iran

**Xiaochang Wang**

China

**Zhiwei Wang**

China

**Dawei Wang**

China

**Rasoul Yarahmadi**

Iran

**Levent Yilmaz**

Turkey

**Masud Yunesian**

Iran

**Ali Zafarzadeh**

Iran

**Paulo Zannin**

Brazil

**Narges Zaredar**

Iran

**Mohammad Ali Zazouli**

Iran

